# What’s in a pollen provision? Using larval provisions to quantify pesticide exposure in *Megachile rotundata* (Hymenoptera: Megachilidae)

**DOI:** 10.1093/ee/nvag023

**Published:** 2026-03-22

**Authors:** Courtney I MacInnis, Lynae P Ovinge, Thomas S Thompson, Shelley E R Hoover

**Affiliations:** Department of Biological Sciences, University of Lethbridge, Lethbridge, AB, Canada; Department of Biological Sciences, University of Lethbridge, Lethbridge, AB, Canada; Alberta Agriculture and Irrigation, Food Safety Branch, Agri-Food Assurance Section, Edmonton, AB, Canada; Department of Biological Sciences, University of Lethbridge, Lethbridge, AB, Canada

**Keywords:** pollen provisions, alfalfa, pesticide residue, exposure risk

## Abstract

The alfalfa leafcutting bee (LCB) (*Megachile rotundata* Fabricius) is a solitary, managed pollinator widely used in North American agriculture to produce alfalfa and hybrid canola seed. Despite its economic importance, and known sensitivity to certain pesticides, little is known about the specific pesticide residues LCBs encounter during pollination, and toxicity data for this species remains limited. To determine what residues LCBs are commonly exposed to, we screened larval LCB provisions from nine alfalfa sites during pollination in southern Alberta for 69 pesticide residues. Eight residues amongst three classes of pesticides were detected including four fungicides (boscalid, cyprodinil, fludioxonil, and pyraclostrobin), three insecticides (chlorpyrifos, cyhalothrin lambda, and deltamethrin), and the herbicide Velpar (hexazinone). Using the residue data, we calculated site-specific hazard quotients (HQ) using *Apis mellifera* L. LD_50_s to provide context for exposure risk. Sites with residues from multiple pesticide classes tended to have higher—though not always significantly higher—HQs than sites with residues from only one class. These findings provide a regional profile for pesticide exposure for LCBs in southern Alberta and identifies compounds of potential concern for future toxicological testing and pollinator management.

## Graphical abstract

**Graphical Abstract:**
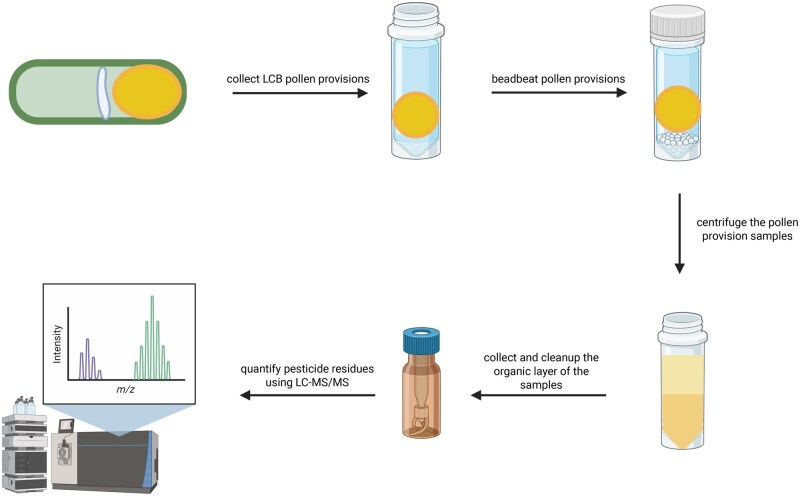

## Introduction

The alfalfa leafcutting bee (LCB) *Megachile rotundata* Fabricius Hymenoptera: Megachilidae is the most intensively managed solitary bee (Pitts-Singer and Cane 2011). In North America LCBs are most often used to pollinate alfalfa (*Medicago sativa* L. Fabales: Fabaceae), leading to a 50% increase in seed production as a result of LCB pollination (Pitts-Singer and Cane 2011). In Canada, LCBs are used to pollinate lowbush blueberries and legume crops and provide about half of the pollination services required to produce hybrid canola seed, contributing over $325 million annually to Canadian agriculture (Canadian Food Inspection Agency 2023). Although LCBs pollinate alfalfa in the United States, the country relies in part on importing Canadian-produced LCBs, which can be costly (Donahoo et al. 2021), to fulfil its alfalfa pollination requirements (Pitts-Singer and Cane 2011, Pitts-Singer 2013). This means LCBs must be healthy enough to ensure effective crop pollination, which is why it is important to understand what pesticides are present in fields where leafcutters are stocked, given that pesticide exposure can negatively affect bee health and survival (Pettis et al. 2013, Siviter et al. 2021, Straub et al. 2021, Walker et al. 2022).

Adult and larval LCBs, like social bees such as honey bees (*Apis mellifera* L. Hymenoptera: Apidae), can be exposed to pesticides through the ingestion of contaminated nectar or pollen (Kopit and Pitts-Singer 2018). However, unlike honey bee larvae, which are fed glandular secretions along with nectar and pollen by nurse bees (Crailsheim 1992), LCB larvae each receive a single mass provision of approximately 90 mg from their mother. This provision consists of 33% to 36% pollen and 64% to 67% nectar by weight (Klostermeyer et al. 1973, Cane et al. 2011). In honey bees, nurse bees can act as biological filters, reducing the overall quantity of pesticides in the food delivered to the larvae (Böhme et al. 2018, Traynor et al. 2021a, Wueppenhorst et al. 2024). In contrast, LCB larvae receive no care from intermediaries—if their provisions are contaminated, the larvae must consume the contaminated food as is to survive (Kopit and Pitts-Singer 2018). In recent years, multiple research groups have investigated the effects of pesticides and chemicals on honey bees, as well as bumble bees, and solitary bees including *Osmia* spp. Their findings indicate that pesticide exposure increases susceptibility to pathogens (Pettis et al. 2013, Siviter et al. 2021), alters immune responses to pathogens (Alaux et al. 2010, Vidau et al. 2011, Aufauvre et al. 2012, Aufauvre et al. 2014, Siviter et al. 2021), decreases longevity (Wu et al. 2011, Henry et al. 2012, Williams et al. 2015, Siviter et al. 2021, Straub et al. 2021, Walker et al. 2022), as well as reproduction (Stuligross and Williams 2020, Stuligross et al. 2023, Albacete et al. 2024, Nicholson et al. 2024), affects learning (Muth et al. 2019), and induces behavioral changes (Eiri and Nieh 2012, Kenna et al. 2019, Stuligross et al. 2023, Keodara et al. 2024). Studies on LCBs have also reported pesticide-related effects, including disrupted nest cavity recognition for female LCBs (Artz and Pitts-Singer 2015), changes to adult longevity and reproduction (Mayer et al. 2001, Hodgson et al. 2011, Gradish et al. 2012, Pitts-Singer and Barbour 2017, Piccolomini et al. 2018, Hayward et al. 2019), and changes in respiration rate (Piccolomini et al. 2018). Additionally, exposure to pesticides as larvae has been linked to reduced longevity (Hodgson et al. 2011, Gradish et al. 2012, Pitts-Singer and Barbour 2017). Notably, LCBs lack specific cytochrome P450 enzymes that belong to or are related to the CYP9Q subfamily, which are known to aid in the detoxification of neonicotinoid and butenolide pesticides in other bee species (Piccolomini et al. 2018, Hayward et al. 2019). As a result, LCBs are significantly more sensitive to certain pesticides that have minimal effects on other bee species, including *A. mellifera* (Hayward et al. 2019), the typical model species for screening non-target effects.

Despite evidence that LCBs are highly sensitive to certain pesticides, limited toxicity data exists for many commonly used pesticides and chemicals. Furthermore, a comprehensive list of pesticides to which LCBs are commonly exposed to is also lacking. This knowledge gap hinders the development of relevant toxicity data that could inform regulatory decisions, including identifying pesticides that are safe to use when LCBs are present. To address this gap and support the Canadian LCB population and the industry, we performed screens for 69 different pesticide residues on LCB pollen provisions collected across nine sites in southern Alberta, an agricultural hub and the primary site for hybrid canola seed production in Canada, and where many of the LCBs exported to the United States are produced. By quantifying pesticide residues found in pollen provisions, we can improve management practices by identifying pesticides that require further testing, or should not be used when LCBs are present.

## Methods

### Sample Collection

Leafcutter bee pollen provisions were collected from LCB shelters (which are typically east-facing structures that hold a series of nest blocks) on irrigated quarter sections containing alfalfa at nine different locations (referred to as locations A through I) in the southern Alberta towns of Monarch, Rainier, and Rosemary on 22 July 2014 and 30 July 2014. All sites were at least 5 km apart, with the majority of sites being 30 to 100 km apart, limiting drift between sampling sites. Leafcutter bees were stocked on fields based on bloom date, which generally ranges from mid-June to mid-July in this area. Pollen provisions were collected by breaking apart Styrofoam nest blocks, taking apart wooden nest blocks, or using forceps to collect pollen provision material from LCB brood cells with eggs from within nest blocks. All pollen provisions used in this study were collected from the same shelter at each location. This sampling resulted in a total of 64 pollen provisions across nine shelters/locations Table 1.

**Table 1.** Number of pollen balls collected at each location

Location	Number of pollen balls collected
**A**	6
**B**	4
**C**	12
**D**	4
**E**	2
**F**	8
**G**	5
**H**	17
**I**	6

### Determination of Pesticide Residues

Single pollen provision samples were analyzed for 69 pesticide residues using liquid chromatography-tandem mass spectrometry (LC-MS/MS). The sample preparation procedure was based on the European Standard EN 15662:2008 methodology for the determination of pesticides in foods of plant origin which was modified to accommodate the reduced sample size. Briefly, individual pollen provisions were processed using a miniaturized QuEChERS method to permit handling of small samples, on average less than 0.1 g, as opposed to the nominal 10 g food samples typically analyzed in other applications. After partitioning of the pesticide residues into acetonitrile, the sample extracts were cleaned using dispersive solid phase extraction. The final extracts were analyzed by LC-MS/MS, which permitted screening for 69 different pesticide residues with limits of quantitation ranging from 5 to 20 ng/g.

Details regarding the sample preparation procedure and the instrumental analysis are provided in the Supplementary Information on the Analytical Methodology for the Determination of Pesticide Residues in Pollen Provision Samples, and Supplementary Table S1.

### Mean Pesticide Concentration

The concentrations of all pesticide residues present in each pollen provision sample at each location were summed and then averaged to calculate the mean pesticide residue concentration for each of the nine locations (Traynor et al. 2021b).

### Mean Pesticide Prevalence

Prevalence (*ie* presence or absence) for each of the eight pesticide residues detected in this study was calculated for each pollen provision sample at each location. Prevalence was then averaged across all samples within a location to determine the mean pesticide residue prevalence for that location.

### Proportion of Samples with Pesticides

Each pollen provision sample at each location was scored for the presence of the three different pesticide classes (fungicide, insecticide, and herbicide) detected in this study. The proportion of samples at each location with each pesticide class present was then calculated.

### Mean Pollen Provision Hazard Quotient

A hazard quotient (HQ) is a risk assessment metric used to evaluate the potential harm of pesticide exposure to organisms, including bees. It is a unitless value that compares the detected pesticide residue levels in an environment (*eg* pollen, nectar, soil) to the known toxicity of that pesticide to a specific species. A pollen provision HQ was calculated for each pollen provision sample at each location by summing all pesticide residue concentrations (in ppb) divided by their respective LD_50_s (in µg/bee) (Stoner and Eitzer 2013, Traynor et al. 2016, Traynor et al. 2021b). Pollen provision HQs were then averaged across all samples within a location to determine the mean pollen provision HQ for that location. Due to the absence of oral acute LD_50_ values for LCBs for all eight pesticides detected in this study, oral acute LD_50_s for *Apis mellifera* adults were used. The one exception was the herbicide Velpar (hexazinone) which had no known oral acute LD_50_ for *A. mellifera*, so an LD_50_ of unknown mode *A. mellifera* was used. All LD_50_ values used in the pollen provision HQ calculations were obtained from the University of Hertfordshire Pesticide Properties DataBase (PPDB, https://sitem.herts.ac.uk/aeru/ppdb/en/index.htm) and can be found in Supplementary Table S2. Assuming that the average weight of a LCB pollen provision is 90 mg (0.090 g)(Cane et al. 2011), and that it is entirely consumed, the pollen provision HQ that would result in a 50% kill dose is 11,111.11:


$$\mathrm{Dose}\;(ng)=\mathrm{Concentr}a\mathrm{tion}\;(\frac{ng}{g})\;\times \;0.090g$$

Concentration × 0.090=LD50 × 1000 (convert to ng from µg)

$$\mathrm{Concentration}\;=\;\frac{LD50\;\times \;1000}{0.090}$$

$$HQ=\;\frac{\mathrm{Concentration}}{LD50}=\;\frac{\left(\frac{LD50\;\times \;1000}{0.090}\right)}{LD50}\;=\;\frac{1000}{0.090}=11,111.11$$

Although pesticides are known to have both antagonistic and synergistic effects on bees (Traynor et al. 2016, Sgolastra et al. 2017, Tosi and Nieh 2019, Tosi et al. 2022), the limited data available for LCBs and the complexities of pesticide interactions necessitates the use of this oversimplified model, which likely underestimates toxicity.

### Mean Pesticides Contributing 55+ Points to Pollen Provision HQ

The number of pesticide residues contributing at least 55 points to a pollen provision HQ score (55 points represents 0.5% of a LCBs LD_50_ under the framework used here) was calculated for each sample at each location. The number of pesticide residues was then averaged by location to determine the mean number of pesticide residues contributing at least 55 points to the pollen provision HQ score at each location.

### Statistical Analyses

All statistical analyses were performed in ‘R’ v. 4.2.1 “Funny Looking Kid” within ‘R’studio v. 2022.07.2 + 576 “Spotted Wakerobin” for MAC OS X.

#### Quantification of Pesticide Concentrations

To determine if there was an effect of location on the concentrations of pesticides found in each pollen provision, Kruskal–Wallis rank sum tests followed by Dunn’s test of multiple comparisons were used (dunn.test, 1.3.5, dunn.test) for seven of the eight pesticides. For the fungicide pyraclostrobin a generalized linear mixed model with a negative binomial distribution was used (glmmTMB, 1.1.7, glmmTMB) as it best fit the data. Model fit was assessed by plotting the scaled residuals, examining Levene’s test for homogeneity of variance, and examining the Kolmogorov–Smirnov’s test for overdispersion (simulateResiduals, 0.4.6, DHARMa). The significance of the predictor variable (location) was evaluated using Anova (Anova, 3.1-0, car). A post-hoc test was completed using emmeans (emmeans, 1.8.5, emmeans) with a Benjamini-Hochberg correction for multiple comparisons.

#### Mean Pesticide Concentration

To examine the effect of location on summed mean pesticide residue concentrations for each pollen provision sample, a generalized linear mixed model with a negative binomial distribution followed by a post-hoc test using emmeans with a Benjamini-Hochberg correction for multiple comparisons was used as above. The significance of the predictor variable (location) and model fit were also assessed as above.

#### Mean Pesticide Prevalence

Pesticide residue prevalence, or the average number of pollen provision samples at each site positive for any pesticide residue, was assessed using a generalized linear model (glm, 1.1-31, lme4) with a binomial distribution. The significance of the predictor variable (location) was evaluated using Anova as above. Model fit and post hoc tests were assessed and completed as above.

##### Proportion of Samples with Pesticides

To determine the proportion of samples with each pesticide class (fungicide, insecticide, and herbicide) present at each location, Kruskal–Wallis rank sum tests followed by Dunn’s test for multiple comparisons were completed for each of the pesticide classes as above.

#### Mean Pollen Provision Hazard Quotient

To examine the effect location had on pollen provision HQs, a Kruskal–Wallis rank sum test followed by Dunn’s test for multiple comparisons was completed as above.

#### Mean Pesticides Contributing 55+ Points to Pollen Provision HQ

Differences in the number of pesticide residues at each location contributing more than 55 points to pollen provision HQs were evaluated using a Kruskal–Wallis rank sum test followed by Dunn’s test for multiple comparisons was completed as above.

## Results

### Quantification of Pesticide Concentrations

**Fig. 1.**
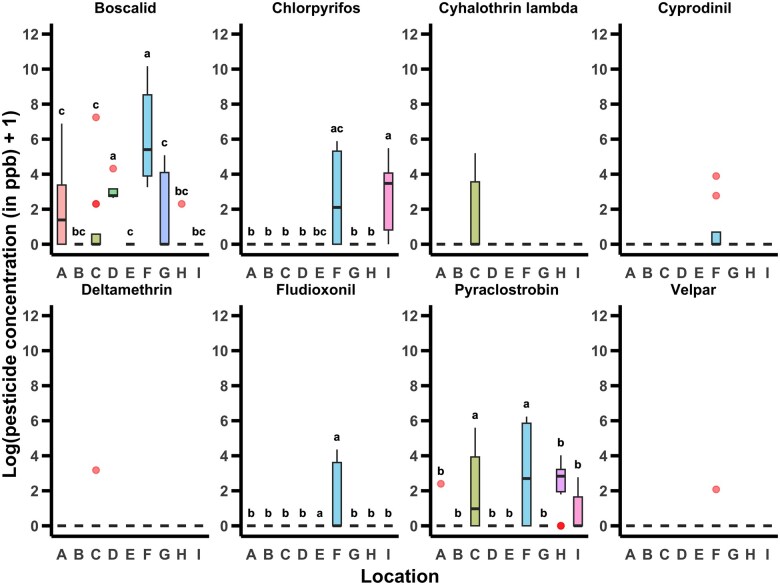
Log_10_-transformed pesticide concentration +1 in ppb for each location. Boxes represent the interquartile range, bars indicate the median, and whiskers span 1.5 × the interquartile range. Red dots represent outliers. Letters above boxes for a given pesticide indicate statistically significant differences (α = 0.05) in pesticide concentration among locations.

We detected eight out of the 69 pesticide residues that we screened for: boscalid, chlorpyrifos, cyhalothrin lambda, cyprodinil, deltamethrin, fludioxonil, pyraclostrobin, and Velpar. There was an effect of location on pesticide residue concentration in collected pollen provisions for five of the eight pesticides detected. There were significantly higher levels of the fungicide boscalid present in pollen provisions collected from locations D and F (χ^2^_8_ = 39.279, *P < *0.001, Fig. 1), while significantly higher levels of the insecticide chlorpyrifos were found in pollen provisions at locations F. The chlorpyrifos concentrations at location F were not significantly different to those found at locations E and I (χ^2^_8_ = 32.34, *P < *0.001, Fig. 1). Concentrations of cyhalothrin lambda were also present at significantly higher levels in pollen provisions at locations B-E, G, and I (χ^2^_8_ = 18.176, *P = *0.020, Fig. 1). Higher levels of the fungicides fludioxonil and pyraclostrobin were found in pollen provisions collected at locations E and F (χ^2^_8_ = 21.674, *P = *0.006, Fig. 1) and C and F (χ^2^_8_ = 20.797, *P = *0.008, Fig. 1), respectively. Finally, there was no effect of location on the concentration of the fungicide cyprodinil (χ^2^_8_ = 14.222, *P = *0.076, Fig. 1), insecticide deltamethrin (χ^2^_8_ = 4.333, *P = *0.826, Fig. 1), or herbicide Velpar (χ^2^_8_ = 7, *P = *0.537, Fig. 1) present in collected pollen provisions.

### Mean Pesticide Concentration

**Fig. 2.**
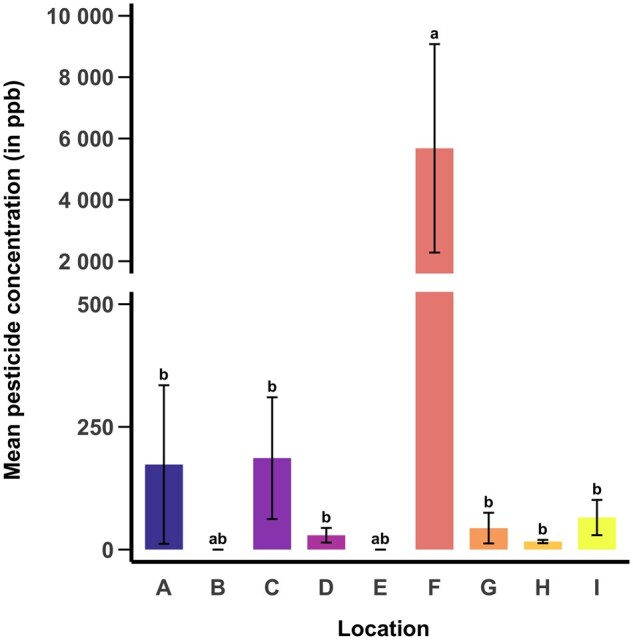
Mean (± SE) summed concentrations of all pesticides at each location. Letters above bars indicate statistically significant differences (α = 0.05) in the average sum of pesticide concentrations among locations.

There was a significant effect of location on mean pesticide residue concentration for all pesticides detected in collected pollen provisions. Location F had an increased mean pesticide residue concentration compared to all locations except B and E (χ^2^_8_ = 55.53, *P <* 0.001, Fig. 2).

### Mean Pesticide Prevalence

**Fig. 3.**
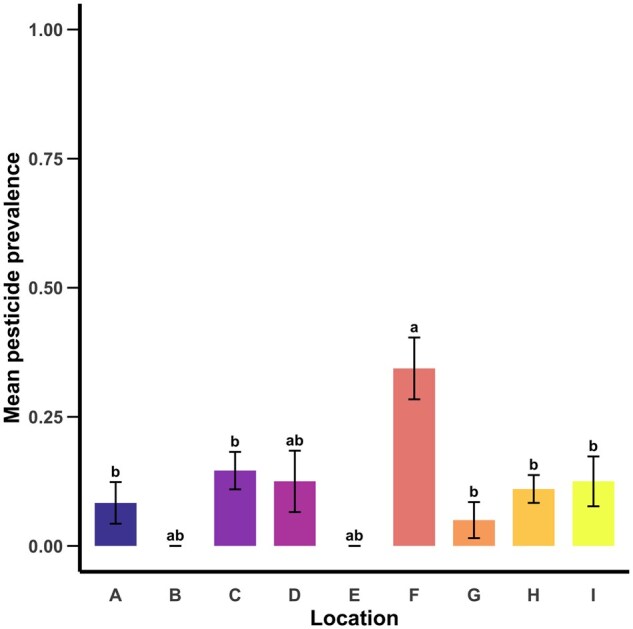
Mean (± SE) pesticide prevalence for each of the nine locations (1.00 = all eight pesticides detected in all samples). Letters above bars indicate statistically significant differences (α = 0.05) in pesticide prevalence among locations.

Pesticide residue prevalence in pollen provisions also differed by location (χ^2^_8_ = 37.083, *P < *0.001, Fig. 3). The highest pesticide residue prevalence was found at location F, which had significantly more pesticide residues present than locations A, C, and G-I but not B, D, and E (see Fig. 3).

### Proportion of Samples with Pesticides

**Fig. 4.**
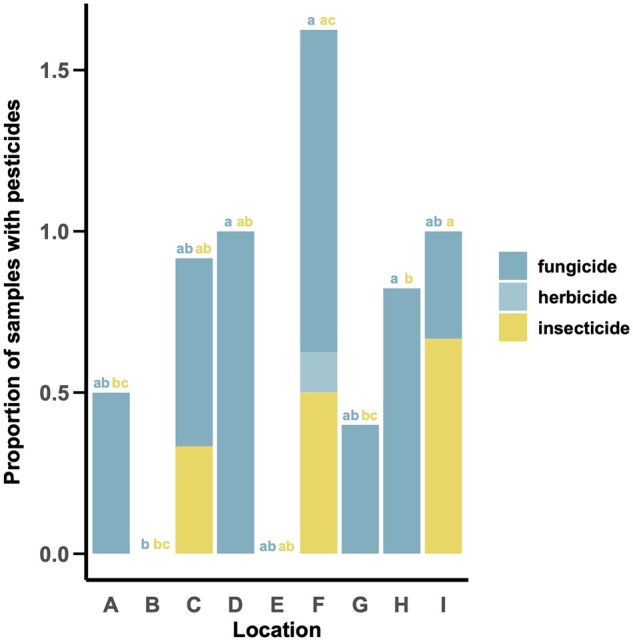
Proportion of samples at each location with a fungicide, herbicide, and/or insecticide detection. Letters above bars in teal indicate statistically significant differences (α = 0.05) in the proportion of samples with fungicides present by location while letters in yellow represent statistically significant differences (α = 0.05) in the proportion of samples with insecticides present by location.

A difference in the proportion of pollen provision samples with fungicides detected by location was detected (χ^2^_8_ = 23.434, *P = *0.002). Locations with the highest proportion of fungicide-containing pollen provisions were D, F, and H (Fig. 4). As there was only one pollen provision sample with an herbicide detected (at location F), there were no differences in the proportion of pollen provision samples with herbicides present among locations (χ^2^_8_ = 7, *P = *0.537, Fig. 4). Finally, location also had an effect on the proportion of pollen provision samples with insecticides present. Location I had a significantly higher proportion of samples with insecticides present compared to locations A, B, G, and H (χ^2^_8_ = 24.231, *P = *0.002, Fig. 4).

### Mean Pollen Provision Hazard Quotient

**Fig. 5.**
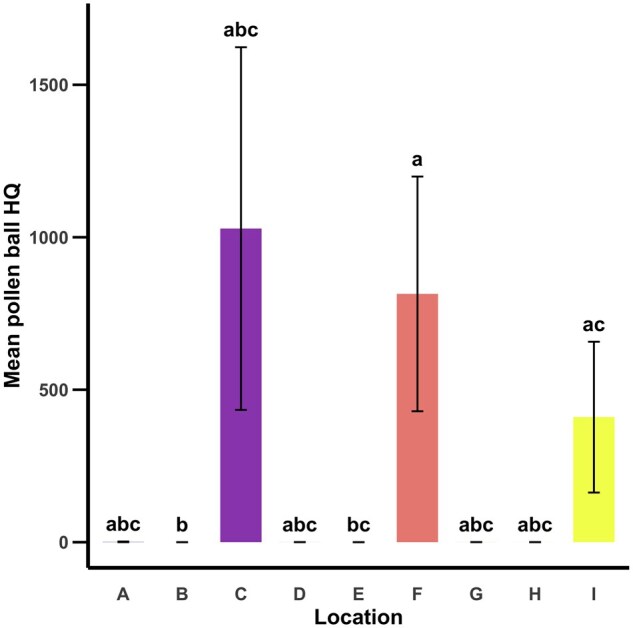
Mean (± SE) HQ per pollen provision at each location. The mean pollen provision HQ is an estimate of a LCB larva’s consumption risk (pesticide residues in ppb/respective LD_50_s in µg/bee). Letters above bars indicate statistically significant differences (α = 0.05) in HQ among locations.

Although location C had the highest mean pollen provision HQ, there were only significant differences in mean pollen provision HQs between locations B, E, and F with location F having a significantly higher mean pollen provision HQ compared to locations B and E (χ^2^_8_ = 22.036, *P = *0.005, Fig. 5).

### Mean Pesticides Contributing 55+ Points to Pollen Provision HQ

**Fig. 6.**
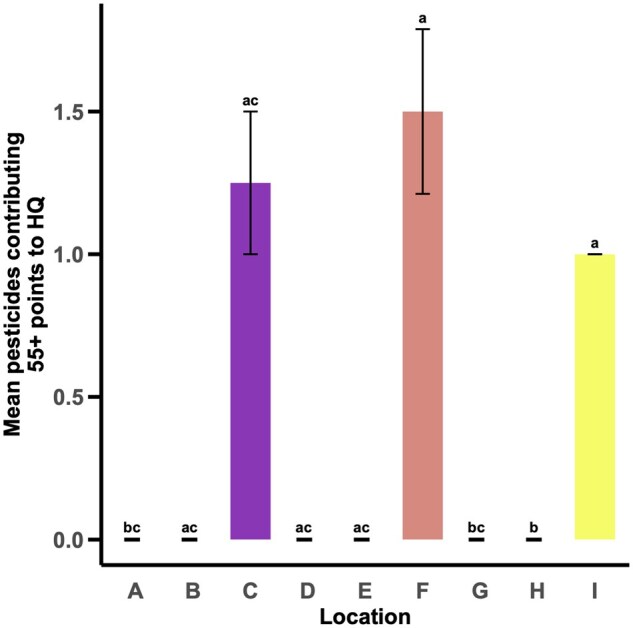
Mean (± SE) number of pesticides contributing more than 55 points to the pollen provision HQ at each location. Letters above bars indicate statistically significant differences (α = 0.05) in the number of pesticides contributing more than 55 points to the pollen provision HQ among treatments.

The number of pesticide residues contributing 55+ points to the pollen provision HQs varied by location (χ^2^_8_ = 25.156, *P *= 0.001, Fig. 6). Location F had the highest number of residues contributing 55+ points to pollen provision HQs followed by locations C and I. Locations F and I had significantly more pesticides contributing 55+ points to their pollen provision HQs than locations A, G, and H (Fig. 6).

## Discussion

To our knowledge, this is the first study to screen for and quantify pesticide residues to which LCBs are exposed to on alfalfa in southern Alberta. We screened LCB pollen provisions collected across nine different locations for 69 residues, detecting and quantifying eight: four fungicides, three insecticides, and one herbicide. The two fungicides with two of the highest oral acute LD_50_s in this study (lower toxicity), boscalid and pyraclostrobin, were the most prevalent and were detected at increased concentrations compared to the six other residues detected in this study. Unsurprisingly, locations with higher mean pesticide residue concentrations also had elevated levels of boscalid and pyraclostrobin, along with a greater proportion of the eight pesticides detected in the samples. Fungicides were the most prevalent pesticide residues found at each of the study locations, followed by insecticides and herbicides. The herbicide detected in this study, Velpar (hexazinone), was found only in one sample at one location.

Generally, the proportion of samples at each location with pesticide residues increased with the prevalence of residue types (fungicides, herbicides, and insecticides) detected. Three of the four locations (C, F, I) with the largest proportion of samples with pesticide residues had at least two types of pesticide residues present (fungicides and insecticides). The location (F) with the highest proportion of samples with pesticide residues had the greatest diversity of pesticides present (fungicides, herbicides, and insecticides). Notably, the three sites (C, F, I) with the greatest diversity of pesticide residues present also had the highest mean pollen provision HQs, and were the only sites with pesticide residues contributing more than 55 points to their HQs. This was largely due to the presence of three insecticides (chlorpyrifos, cyhalothrin lambda, deltamethrin) that all have low oral acute LD_50_s (high toxicity). Location C had a mean pollen provision HQ of 1,028.45, which means that LCBs consuming pollen provisions at this site were consuming nearly 10% of their LD_50_s—the dose at which 50% of individuals die. Locations F and I had mean pollen provision HQs of 814.18 and 410.03, respectively which means that LCBs at these two sites were consuming 7.31 and 3.69% of their LD_50_s. It is important to note that consuming even a portion of an LD_50_ could increase the potential for negative or sublethal consequences. Some of the pesticide residues detected in this study have been associated with such sublethal effects in adult *A. mellifera* workers, including decreased wingbeat frequency (Liao et al. 2019), reduced pollen consumption and protein digestion (Degrandi-Hoffman et al. 2015), decreased responsiveness to sucrose (Li et al. 2017), impaired learning and memory (Vandame et al. 1995, Li et al. 2017, Liao et al. 2019, Zhang et al. 2020), reduced fecundity (Dai et al. 2010), and disrupted waggle dance communication (Zhang et al. 2020). Although location C had the highest mean pollen provision HQ out of all nine locations, it was location F that had the most pesticides contributing 55+ points to its hazard quotient due to the presence of the herbicide, Velpar. Although the method for quantifying pollen provision HQs in this study is imperfect and oversimplified (Stoner and Eitzer 2013, Traynor et al. 2016, Traynor et al. 2021b)—particularly due to limited data on LCBs, the complexity of pesticide interactions, and the absence of nectar residue data (Thompson 2021)—it still allows us to identify what residues are present and may pose risks to LCB health.

The main purpose of this study was to identify the pesticides present in pollen provisions in southern Alberta to inform management recommendations. Of the eight compounds that we were able to identify and quantify from pollen provisions collected in the region, none had any LD_50_ values (acute or chronic, oral or contact) available for LCBs. As a result, oral acute LD_50_s and unknown mode LD_50_s for adult *A. mellifera* were used in all toxicity calculations. This approach presents several limitations due to key differences between honey bees and LCBs, including variations in life history strategies, routes of exposure, and detoxification mechanisms (Crailsheim 1992, Pitts-Singer and Cane 2011, Kopit and Pitts-Singer 2018, Piccolomini et al. 2018, Hayward et al. 2019, Sgolastra et al. 2019). Of particular concern are the differences in larval feeding strategies which may significantly influence pesticide exposure and risk and remain largely uncharacterized for both honey bees and LCBs.

Honey bee larvae are fed brood food, a liquid composed of glandular secretions from nurse bees, honey, and bee bread (processed pollen of various sources, honey, enzymes) (Winston 1991). The honey and bee bread used in brood food are often stored for extended periods of time and undergo complex aging and enzymatic transformations before being processed by nurse bees and fed to larvae (Sgolastra et al. 2019). Pesticide levels in brood food may be further reduced via dilution, as foragers collect nectar and pollen from a wide variety of floral sources (Berenbaum and Johnson 2015), sometimes traveling more than 8000 m from the colony (Visscher and Seeley 1982). In contrast, LCB larvae are provisioned with a pollen ball comprised of recently collected and unprocessed (often source-limited [Killewald et al. 2019]) pollen and nectar for consumption. Because LCBs are often placed in agricultural fields for pollination and have a more limited flight range than honey bees (Pankiw and Siemens 1974, Peterson et al. 2006), they have fewer options to dilute pesticide levels in larval food. The different collection and feeding strategies used by LCBs may influence how pesticides are diluted, degraded, or retained in larval food, ultimately affecting exposure levels and toxicological outcomes.

Based on this study, the pesticides to which LCBs are most exposed in southern Alberta are the fungicides boscalid and pyraclostrobin, and the insecticides chlorpyrifos and cyhalothrin lambda. Given that no toxicity data exists for LCBs for any of these pesticides, the insecticides chlorpyrifos and cyhalothrin lambda should be prioritized for further evaluation due to their low oral acute LD_50_ values in adult honey bees (0.15 and 0.027 µg/bee, respectively). Although some sources of variability in our study likely reflect differences in grower management practises (including chemicals used and the timing of their application to agricultural fields), all four pesticides detected have relatively short residual half-lives (RL_50_s) ranging from 3.0 to 8.0 d in and on plant matrices (PPDB, https://sitem.herts.ac.uk/aeru/ppdb/en/index.htm). Consequently, potential negative effects of these pesticides on LCBs could be minimized through improved communication between growers and LCB producers regarding the presence and activity of LCBs, the timing of pesticide applications, as well as what pesticides are being applied, and what, if any, adjuvants are being used. Synergistic interactions between pesticides have been observed in honey bees (Pilling et al. 1995, Vandame and Belzunces 1998, Johnson et al. 2013, Walker et al. 2022), and increased toxicity has also been reported when certain pesticides are combined with specific adjuvants (Shannon et al. 2023). Improved management practices could include applying pesticides before LCBs are placed on agricultural fields, applying at night or before bloom, and using pesticides or pesticide-adjuvant combinations that have low toxicity to LCBs.

## Supplementary Material

nvag023_Supplementary_Data

## References

[nvag023-B1] Alaux C , Brunet JL, Dussaubat C, et al 2010. Interactions between *Nosema* microspores and a neonicotinoid weaken honey bees (*Apis mellifera*). Environ. Microbiol. 12:774–782. 20050872 10.1111/j.1462-2920.2009.02123.x PMC2847190

[nvag023-B2] Albacete S , Sancho G, Azpiazu C, et al 2024. Exposure to sublethal levels of insecticide-fungicide mixtures affect reproductive success and population growth rates in the solitary bee *Osmia cornuta*. Environ. Int. 190:108919. 39094406 10.1016/j.envint.2024.108919

[nvag023-B3] Artz DR , Pitts-Singer TL. 2015. Effects of fungicide and adjuvant sprays on nesting behavior in two managed solitary bees, *Osmia lignaria* and *Megachile rotundata*. PLoS One 10:e0135688. 26274401 10.1371/journal.pone.0135688 PMC4537283

[nvag023-B4] Aufauvre J , Biron DG, Vidau C, et al 2012. Parasite-insecticide interactions: a case study of *Nosema ceranae* and fipronil synergy on honeybee. Sci. Rep. 2:326. 22442753 10.1038/srep00326 PMC3310228

[nvag023-B5] Aufauvre J , Misme-Aucouturier B, Vigues B, et al 2014. Transcriptome analyses of the honeybee response to Nosema ceranae and insecticides. PLoS One 9:e91686. 24646894 10.1371/journal.pone.0091686 PMC3960157

[nvag023-B6] Berenbaum MR , Johnson RM. 2015. Xenobiotic detoxification pathways in honey bees. Curr. Opin. Insect Sci. 10:51–58. 29588014 10.1016/j.cois.2015.03.005

[nvag023-B7] Böhme F , Bischoff G, Zebitz CPW, et al 2018. From field to food—will pesticide-contaminated pollen diet lead to a contamination of royal jelly? Apidologie 49:112–119.

[nvag023-B8] Canadian Food Inspection Agency, Animal Biosecurity: Alfalfa leafcutting bee producer guide to the National Bee Farm Level Biosecurity Standard 2023. https://inspection.canada.ca/en/animal-health/terrestrial-animals/biosecurity/standards-and-principles/alfalfa-leaf-cutting-bee [accessed 4 April 2025].

[nvag023-B9] Cane JH , Gardner DR, Harrison PA. 2011. Nectar and pollen sugars constituting larval provisions of the alfalfa leaf-cutting bee (*Megachile rotundata*) (Hymenoptera: Apiformes: Megachilidae). Apidologie 42:401–408.

[nvag023-B10] Crailsheim K. 1992. The flow of jelly within a honeybee colony. J. Comp. Physiol. B 162:681–689.

[nvag023-B11] Dai P-L , Wang Q, Sun J-H, et al 2010. Effects of sublethal concentrations of bifenthrin and deltamethrin on fecundity, growth, and development of the honeybee *Apis mellifera ligustica*. Environ. Toxicol. Chem. 29:644–649. 20821489 10.1002/etc.67

[nvag023-B12] Degrandi-Hoffman G , Chen Y, Dejong EW, et al 2015. Effects of oral exposure to fungicides on honey bee nutrition and virus levels. J. Econ. Entomol. 108:2518–2528. 26318004 10.1093/jee/tov251

[nvag023-B13] Donahoo CK , O’Neill KM, Delphia CM, et al 2021. Mortality dynamics and life tables of *Megachile rotundata* (Hymenoptera: Megachilidae), a pollinator managed for alfalfa seed production. Environ. Entomol. 50:444–454. 33439970 10.1093/ee/nvaa176

[nvag023-B14] Eiri DM , Nieh JC. 2012. A nicotinic acetylcholine receptor agonist affects honey bee sucrose responsiveness and decreases waggle dancing. J. Exp. Biol. 215:2022–2029. 22623190 10.1242/jeb.068718

[nvag023-B15] Gradish AE , Scott-Dupree CD, Cutler GC. 2012. Susceptibility of *Megachile rotundata* to insecticides used in wild blueberry production in Atlantic Canada. J. Pest Sci. 85:133–140.

[nvag023-B16] Hayward A , Beadle K, Singh KS, et al 2019. The leafcutter bee, *Megachile rotundata*, is more sensitive to N-cyanoamidine neonicotinoid and butenolide insecticides than other managed bees. Nat. Ecol. Evol. 3:1521–1524. 31666734 10.1038/s41559-019-1011-2

[nvag023-B17] Henry M , Béguin M, Requier F, et al 2012. A common pesticide decreases foraging success and survival in honey bees. Science 336:348–350. 22461498 10.1126/science.1215039

[nvag023-B18] Hodgson EW , Pitts-Singer TL, Barbour JD. 2011. Effects of the insect growth regulator, novaluron on immature alfalfa leafcutting bees, *Megachile rotundata*. J. Insect Sci. 11:43. 21539417 10.1673/031.011.0143 PMC3281451

[nvag023-B19] Johnson RM , Dahlgren L, Siegfried BD, et al 2013. Acaricide, fungicide and drug interactions in honey bees (*Apis mellifera*). PLoS One 8:e54092. 23382869 10.1371/journal.pone.0054092 PMC3558502

[nvag023-B20] Kenna D , Cooley H, Pretelli I, et al 2019. Pesticide exposure affects flight dynamics and reduces flight endurance in bumblebees. Ecol. Evol. 9:5637–5650. 31160987 10.1002/ece3.5143 PMC6540668

[nvag023-B21] Keodara A , Jeker L, Straub L, et al 2024. Novel fungicide and neonicotinoid insecticide impair flight behavior in pollen foraging honey bees, *Apis mellifera*. Sci. Rep. 14:22865. 39354118 10.1038/s41598-024-73235-9 PMC11445536

[nvag023-B22] Killewald MF , Rowe LM, Graham KK, et al 2019. Use of Nest and Pollen Resources by Leafcutter Bees, Genus *Megachile* (Hymenoptera: Megachilidae) in Central Michigan. The Great Lakes Entomol. 52:34–44.

[nvag023-B23] Klostermeyer EC , Mech SJ, Rasmussen WB. 1973. Sex and weight of *Megachile rotundata* (Hymenoptera: Megachilidae) progeny associated with provision weights. J. Kansas Entomol. Soc. 46:536–548.

[nvag023-B24] Kopit AM , Pitts-Singer TL. 2018. Routes of pesticide exposure in solitary, cavity-nesting bees. Environ Entomol 47:499–510.

[nvag023-B25] Li Z , Li M, Huang J, et al 2017. Effects of sublethal concentrations of chlorpyrifos on olfactory learning and memory performances in two bee species, *Apis mellifera* and *Apis cerana*. Sociobiol. 64:174–181.

[nvag023-B26] Liao L-H , Wu W-Y, Dad A, et al 2019. Fungicide suppression of flight performance in the honeybee (*Apis mellifera*) and its amelioration by quercetin. Proc. Biol. Sci. 286:20192041. 31847772 10.1098/rspb.2019.2041 PMC6939911

[nvag023-B27] Mayer D , Kovacs G, Brett B, et al 2001. The effects of spinosad insecticide to adults of *Apis mellifera*, *Megachile rotundata* and *Nomia melanderi* (Hymenoptera: Apidae). Int. J. Hortic. Sci. 7:93–97.

[nvag023-B28] Muth F , Francis JS, Leonard AS. 2019. Modality-specific impairment of learning by a neonicotinoid pesticide. Biol. Lett. 15:20190359. 31362607 10.1098/rsbl.2019.0359 PMC6684981

[nvag023-B29] Nicholson CC , Knapp J, Kiljanek T, et al 2024. Pesticide use negatively affects bumble bees across European landscapes. Nature 628:355–358. 38030722 10.1038/s41586-023-06773-3 PMC11006599

[nvag023-B30] Pankiw P , Siemens B. 1974. Management of *Megachile rotundata* in northwestern Canada for population increase. Can. Entomol. 106:1003–1008.

[nvag023-B31] Peterson JH , Roitberg BD, Peterson JH. 2006. Impacts of flight distance on sex ratio and resource allocation to offspring in the leafcutter bee. Behav. Ecol. Sociobiol. 59:589–596.

[nvag023-B32] Pettis JS , Lichtenberg EM, Andree M, et al 2013. Crop pollination exposes honey bees to pesticides which alters their susceptibility to the gut pathogen *Nosema ceranae*. PLoS One 8:e70182. 23894612 10.1371/journal.pone.0070182 PMC3722151

[nvag023-B33] Piccolomini AM , Whiten SR, Flenniken ML, et al 2018. Acute toxicity of permethrin, deltamethrin, and etofenprox to the alfalfa leafcutting bee. J. Econ. Entomol. 111:1001–1005. 29444244 10.1093/jee/toy014

[nvag023-B34] Pilling ED , Bromleychallenor KAC, Walker CH, et al 1995. Mechanism of synergism between the pyrethroid insecticide λ-cyhalothrin and the imidazole fungicide prochloraz, in the honeybee (*Apis mellifera* L.). Pestic. Biochem. Physiol. 51:1–11.

[nvag023-B35] Pitts-Singer TL. 2013. Intended release and actual retention of alfalfa leafcutting bees (Hymenoptera: Megachilidae) for pollination in commercial alfalfa seed fields. J. Econ. Entomol. 106:576–586. 23786042 10.1603/ec12416

[nvag023-B36] Pitts-Singer TL , Barbour JD. 2017. Effects of residual novaluron on reproduction in alfalfa leafcutting bees, *Megachile rotundata* F. (Megachilidae). Pest Manag. Sci. 73:153–159. 27405042 10.1002/ps.4356

[nvag023-B37] Pitts-Singer TL , Cane JH. 2011. The alfalfa leafcutting bee, *Megachile rotundata*: the world’s most intensively managed solitary bee. Annu. Rev. Entomol. 56:221–237. 20809804 10.1146/annurev-ento-120709-144836

[nvag023-B38] Sgolastra F , Hinarejos S, Pitts-Singer TL, et al 2019. Pesticide exposure assessment paradigm for solitary bees. Environ. Entomol. 48:22–35. 30508080 10.1093/ee/nvy105

[nvag023-B39] Sgolastra F , Medrzycki P, Bortolotti L, et al 2017. Synergistic mortality between a neonicotinoid insecticide and an ergosterol-biosynthesis-inhibiting fungicide in three bee species. Pest Manag. Sci. 73:1236–1243. 27685544 10.1002/ps.4449

[nvag023-B40] Shannon B , Walker E, Johnson RM. 2023. Toxicity of spray adjuvants and tank mix combinations used in almond orchards to adult honey bees (*Apis mellifera*). J. Econ. Entomol. 116:1467–1480. 37656894 10.1093/jee/toad161 PMC10564267

[nvag023-B41] Siviter H , Bailes EJ, Martin CD, et al 2021. Agrochemicals interact synergistically to increase bee mortality. Nature 596:389–392. 34349259 10.1038/s41586-021-03787-7

[nvag023-B42] Stoner KA , Eitzer BD. 2013. Using a hazard quotient to evaluate pesticide residues detected in pollen trapped from honey bees (*Apis mellifera*) in Connecticut. PLoS One 8:e77550. 24143241 10.1371/journal.pone.0077550 PMC3797043

[nvag023-B43] Straub L , Villamar-Bouza L, Bruckner S, et al 2021. Negative effects of neonicotinoids on male honeybee survival, behaviour and physiology in the field. J. Appl. Ecol. 58:2515–2528.

[nvag023-B44] Stuligross C , Melone GG, Wang L, et al 2023. Sublethal behavioral impacts of resource limitation and insecticide exposure reinforce negative fitness outcomes for a solitary bee. Sci. Total Environ. 867:161392. 36621507 10.1016/j.scitotenv.2023.161392

[nvag023-B45] Stuligross C , Williams NM. 2020. Pesticide and resource stressors additively impair wild bee reproduction. Proc. R Soc. B 287:20201390. 10.1098/rspb.2020.1390 PMC7542814 32993468

[nvag023-B46] Thompson HM. 2021. The use of the Hazard Quotient approach to assess the potential risk to honeybees (*Apis mellifera*) posed by pesticide residues detected in bee-relevant matrices is not appropriate. Pest Manag. Sci. 77:3934–3941. 33899320 10.1002/ps.6426

[nvag023-B47] Tosi S , Nieh JC. 2019. Lethal and sublethal synergistic effects of a new systemic pesticide, flupyradifurone (Sivanto(R)), on honeybees. Proc. R Soc. B 286:20190433. 10.1098/rspb.2019.0433 PMC6501679 30966981

[nvag023-B48] Tosi S , Sfeir C, Carnesecchi E, et al 2022. Lethal, sublethal, and combined effects of pesticides on bees: a meta-analysis and new risk assessment tools. Sci. Total Environ. 844:156857. 35760183 10.1016/j.scitotenv.2022.156857

[nvag023-B49] Traynor KS , Pettis JS, Tarpy DR, et al 2016. In-hive pesticide exposome: assessing risks to migratory honey bees from in-hive pesticide contamination in the Eastern United States. Sci. Rep. 6:33207. 27628343 10.1038/srep33207 PMC5024099

[nvag023-B50] Traynor KS , Tosi S, Rennich K, et al 2021b. Pesticides in honey bee colonies: establishing a baseline for real world exposure over seven years in the USA. Environ. Pollut. 279:116566. 33839524 10.1016/j.envpol.2021.116566

[nvag023-B51] Traynor KS , vanEngelsdorp D, Lamas ZS. 2021a. Social disruption: sublethal pesticides in pollen lead to *Apis mellifera* queen events and brood loss. Ecotoxicol. Environ. Saf. 214:112105. 33690003 10.1016/j.ecoenv.2021.112105

[nvag023-B52] van dame R , Meled M, Colin ME, et al 1995. Alteration of the homing‐flight in the honey bee *Apis mellifera* L. Exposed to sublethal dose of deltamethrin. Environ. Toxicol. Chem. 14:855–860.

[nvag023-B53] Vandame R , Belzunces LP. 1998. Joint actions of deltamethrin and azole fungicides on honey bee thermoregulation. Neurosci. Lett. 251:57–60. 9714464 10.1016/s0304-3940(98)00494-7

[nvag023-B54] Vidau C , Diogon M, Aufauvre J, et al 2011. Exposure to sublethal doses of fipronil and thiacloprid highly increases mortality of honeybees previously Infected by *Nosema ceranae*. PLoS One 6:e21550. 21738706 10.1371/journal.pone.0021550 PMC3125288

[nvag023-B55] Visscher PK , Seeley TD. 1982. Foraging strategy of honeybee colonies in a temperate deciduous forest. Ecol. 63:1790–1801.

[nvag023-B56] Walker EK , Brock GN, Arvidson RS, et al 2022. Acute toxicity of fungicide–insecticide–adjuvant combinations applied to almonds during bloom on adult honey bees. Environ. Toxicol. Chem. 41:1042–1053. 35060643 10.1002/etc.5297 PMC9313819

[nvag023-B57] Williams GR , Troxler A, Retschnig G, et al 2015. Neonicotinoid pesticides severely affect honey bee queens. Sci. Rep. 5:14621. 26459072 10.1038/srep14621 PMC4602226

[nvag023-B58] Winston ML. 1991. The biology of the honey bee. Harvard University Press.

[nvag023-B59] Wu JY , Anelli CM, Sheppard WS. 2011. Sub-lethal effects of pesticide residues in brood comb on worker honey bee (*Apis mellifera*) development and longevity. PLoS One 6:e14720. 21373182 10.1371/journal.pone.0014720 PMC3044129

[nvag023-B60] Wueppenhorst K , Alkassab AT, Beims H, et al 2024. Nurse honey bees filter fungicide residues to maintain larval health. Curr. Biol. 34:5570–5577.e11. 39476835 10.1016/j.cub.2024.10.008

[nvag023-B61] Zhang ZY , Li Z, Huang Q, et al 2020. Deltamethrin impairs honeybees (*Apis mellifera*) dancing communication. Arch. Environ. Contam. Toxicol. 78:117–123. 31642948 10.1007/s00244-019-00680-3

