# Supplementary material for: What’s in a pollen provision? Using larval provisions to quantify pesticide exposure in Megachile rotundata (Hymenoptera: Megachilidae)
Source: Environ Entomol. 2026 Mar 22;55(2):nvag023. doi: 10.1093/ee/nvag023 (PMC13017921; doi:10.1093/ee/nvag023)
Supplement: nvag023_Supplementary_Data [file nvag023_supplementary_data.zip › 17-Mar-2026_113512_Supplementary_Information_for_LCB_pesticide_residue_MS.docx]

**Supplementary Information on the Analytical Methodology for the Determination of Pesticide Residues in Pollen Ball Samples**

**Reagents and materials**

Organic solvents acetonitrile and methanol were HPLC grade and suitable for residue analysis (Caledon Laboratories Ltd., Georgetown, ON, Canada). Reagent water (18 MΩ resistivity) was prepared using a NANOpure Diamond water purification system (Barnstead Thermolyne Corporation, Dubuque, IA, USA). LC mobile phase additives ACS reagent grade ammonium formate and formic acid (≥95% purity) were purchased from Caledon Laboratories Ltd. and Sigma-Aldrich Canada Co. (Oakville, ON, Canada), respectively. Individual pesticide standard reference materials were obtained from Sigma-Aldrich Canada and Canadian Life Science Inc. (Peterborough, ON, Canada). Deuterated malathion-d6 was purchased from C/D/N Isotopes Inc. (Point-Claire, QB, Canada).

Two millilitre polypropylene tubes with polypropylene screw caps and ethylene propylene diene monomer rubber o-rings were purchased from Omni International (Kennesaw, GA, USA, part number 19-649S). A mixture of QuEChERS salts consisting of 80 mg of magnesium sulphate, 20 mg of sodium chloride, 20 mg of sodium citrate tribasic dihydrate, and 10 mg of sodium citrate dibasic sesquihydrate were obtained in pre-packaged 2-mL centrifuge tubes (Chromatographic Specialties, Brockville, ON, Canada, part number UCTECQUU22CT). Pre-packaged 2-mL centrifuge tubes containing 150 mg magnesium sulphate, 50 mg of primary-secondary amine (PSA), and 50 mg of endcapped octadecylsilane (C18) adsorbents were also purchased from Chromatographic Specialties (part number UCTCUMPSC18CT).

**Preparation of matrix-matched calibration standards**

Individual stock standard solutions containing 1000 µg/mL of analyte were prepared in acetonitrile. These individual standard solutions were used to prepare a mixed intermediate containing each of the analytes at a concentration of 10 µg/mL in acetonitrile. The intermediate standard solution was subsequently diluted to prepare a working standard solution with individual analyte concentrations of 0.1 µg/mL in acetonitrile.

Pollen which was found to be free of detectable residues of the 69 targeted pesticides was used in the preparation of matrix-matched calibration standards. A 1.3 g sample of homogenized blank pollen was dissolved in 10 mL of reagent water by vortex mixing. Seven 0.75-mL aliquots of the pollen solution were pipetted into 2-mL polypropylene tubes. Six of the tubes were spiked with incremental volumes of the 0.1 µg/mL pesticide standard solution (5, 10, 25, 50, 75, and 100 µL) while the seventh tube was unspiked and served as a negative control sample.

**Sample preparation procedure**

Individual pollen ball samples were weighed into 2-mL polypropylene tubes which were capped and stored at -20 °C pending processing. The samples were extracted using a modified QuEChERS (Quick Easy Cheap Effective Rugged Safe) procedure which was miniaturized to accommodate the small sample size. Each matrix-matched calibration standard or pollen ball sample was fortified with 10 µL of a standard solution containing 1 µg/mL of deuterated malathion-d6 in acetonitrile. Reagent water (0.75 mL) was added to each tube containing a pollen ball sample. A 0.75-mL aliquot of acetonitrile was pipetted into all tubes containing either a matrix-matched calibration standard or pollen ball sample. The tubes were then capped and manually mixed briefly. The contents of a centrifuge tube containing the QuEChERS salt mixture was then added to each tube containing a matrix-matched calibration standard or a pollen ball sample. The individual tubes were briefly shaken by hand immediately after addition of the QuEChERS salt mixture to minimize clumping of the solid material. Once the initial preparation had been carried out on all tubes, they were mixed at high oscillatory speed for one minute using an Omni Bead Ruptor 12 bead mill homogenizer. The tubes were then centrifuged at 3000 rpm for five minutes to facilitate separation of the aqueous and organic phases. The top organic layer from each tube was transferred to a 2-mL centrifuge tube containing magnesium sulphate, PSA, and C18 adsorbents to perform a dispersive solid phase extraction (DSPE) cleanup step. After 5 minutes of mixing using a rocking shaker, the DSPE tubes were centrifuged for five minutes at 3000 rpm. A portion of each cleaned extract was transferred to a 300 µL glass insert held within a 2-mL LC autosampler vial.

**Determination of pesticide residues by LC-MS/MS**

The instrumental analysis was performed using an LC-MS/MS system which consisted of a Shimadzu liquid chromatograph (including a SIL30AC autosampler, dual LC30AD pumps, a CTO30A column oven, and a CBM20A controller module) interfaced to a Sciex 4500 tandem quadrupole mass spectrometer. The chromatographic separation was achieved using a Kinetex pentafluorophenyl column (100 x 2.1 mm i.d. with 1.7 µm particles; part number 00D-4476-AN) protected by a SecurityGuard ULTRA cartridge (part number AJ0-8787), both of which were purchased from Phenomenex (Torrance, CA, USA). The LC mobile phases consisted of 5 mM ammonium formate with 0.1% (v/v) formic acid in water as mobile phase A and 5 mM ammonium formate with 0.1% (v/v) formic acid in methanol as mobile phase B. All analyses were performed with a binary gradient elution program where the initial composition of 2% B was incremented linearly to 100% B over 15 minutes, with the final composition held for an additional 5 minutes. After each analytical run, the mobile phase composition was returned to 2% B and the system was allowed to re-equilibrate for 5 minutes before the next injection. The mobile phase flow rate was kept at 0.3 mL/min throughout the gradient program. The LC column was maintained at 40 °C and an injection volume of 5 µL was used for all analyses.

The Sciex 4500 MS/MS system was equipped with a Turbo V ion source and a TurboIonSpray probe which was operated in the positive electrospray ionization mode. The source temperature was set to 500 °C and the electrospray capillary voltage was 3.5 kV. The nebulizer gas GS1 and the source heater gas GS2 were both set at 30 psi while the curtain gas was maintained at 20 psi. The collisionally activated dissociation cell gas value was set at 8. High purity nitrogen for the source and collision cell gases was provided by a Parker Balston model 75-880 nitrogen generator system. Detection of the pesticides was achieved using multiple reaction monitoring (MRM). Two precursor-to-product ion pairs were monitored for each compound, with one transition used for quantitation and the second used for confirmation of analyte identity. The MS/MS was operated in the scheduled MRM mode where a detection window of ±0.75 min was centred on the retention time of each analyte. The MRM parameters are provided in the table below. Quantitation was performed by external standardization using the matrix-matched calibration standards. The weight of each individual pollen ball sample was taken into consideration when calculating the concentration of any detected pesticide. The malathion-d6 surrogate standard was used to monitor method performance (i.e., extraction and cleanup efficiency).

**Supplementary Table 1.** LC-MS/MS determination of pesticides in pollen ball samples

|  |  |  | **Quantitation MRM Transition** | | **Confirmation MRM Transition** | |  |
| --- | --- | --- | --- | --- | --- | --- | --- |
| **Compound** | **RT (min)** | **DP (V)** | **Precursor > Product (Da)** | **CE (eV)** | **Precursor > Product (Da)** | **CE (eV)** | **LOQ (ng/g)** |
| Acephate | 0.9 | 40 | 184 > 143 | 20 | 184 > 95 | 30 | 10 |
| Atrazine | 10.3 | 50 | 216 > 174 | 24 | 216 > 104 | 36 | 5 |
| Azinphos-methyl | 11.7 | 50 | 318 > 160 | 12 | 318 > 132 | 20 | 5 |
| Azoxystrobin | 11.9 | 60 | 404 > 372 | 16 | 404 > 344 | 34 | 5 |
| Bentazon | 9.3 | 60 | 241 > 199 | 16 | 241 > 107 | 36 | 20 |
| Boscalid | 11.7 | 100 | 343 > 307 | 29 | 343 > 140 | 29 | 5 |
| Bromacil | 9.3 | 60 | 261 > 205 | 20 | 261 > 188 | 38 | 10 |
| Carbaryl | 9.9 | 50 | 202 > 145 | 28 | 202 > 127 | 36 | 5 |
| Carbofuran | 9.2 | 100 | 222 > 165 | 14 | 222 > 123 | 22 | 5 |
| Carboxin | 9.7 | 50 | 236 > 143 | 21 | 236 > 87 | 30 | 5 |
| Chlorantraniliprole | 11.1 | 60 | 484 > 453 | 24 | 484 > 286 | 19 | 5 |
| Chlorpyrifos | 13.8 | 70 | 350 > 97 | 44 | 350 > 198 | 25 | 5 |
| Coumaphos | 13.9 | 70 | 363 > 227 | 34 | 363 > 307 | 24 | 5 |
| Cyfluthrin | 15.1 | 50 | 451 > 191 | 20 | 451 > 434 | 12 | 10 |
| Cyhalothrin lambda | 15.3 | 50 | 467 > 225 | 23 | 467 > 141 | 58 | 10 |
| Cymoxanil | 8.6 | 50 | 199 > 128 | 12 | 199 > 111 | 26 | 5 |
| Cypermethrin | 15.0 | 50 | 433 > 191 | 20 | 433 > 127 | 38 | 10 |
| Cyprodinil | 12.9 | 60 | 226 > 93 | 48 | 226 > 108 | 34 | 10 |
| Deltamethrin | 15.0 | 70 | 523 > 281 | 21 | 523 > 181 | 46 | 10 |
| Diazinon | 13.0 | 50 | 305 > 97 | 42 | 305 > 153 | 25 | 5 |
| Dibrom | 10.7 | 40 | 398 > 127 | 24 | 398 > 109 | 50 | 20 |
| Difenoconazole | 13.5 | 70 | 406 > 251 | 32 | 406 > 337 | 24 | 5 |
| Dimethoate | 7.4 | 50 | 230 > 199 | 10 | 230 > 125 | 22 | 5 |
| Dimethomorph | 12.4 | 60 | 388 > 301 | 30 | 388 > 165 | 40 | 5 |
| Diuron | 11.0 | 50 | 233 > 72 | 32 | 233 > 46 | 32 | 5 |
| EPTC | 11.9 | 40 | 190 > 128 | 15 | 190 > 86 | 17 | 10 |
| Ethofumesate | 11.6 | 50 | 304 > 121 | 27 | 304 > 241 | 16 | 5 |
| Famoxadone | 13.1 | 40 | 392 > 331 | 12 | 392 > 238 | 23 | 5 |
| Fenamidone | 10.9 | 40 | 312 > 92 | 35 | 312 > 236 | 19 | 5 |
| Flucarbazone Na | 9.4 | 40 | 414 > 130 | 20 | 414 > 115 | 50 | 5 |
| Fludioxonil | 12.2 | 40 | 266 > 229 | 16 | 266 > 158 | 40 | 5 |
| Flumioxazin | 11.3 | 50 | 372 > 327 | 31 | 372 > 299 | 42 | 10 |
| Fluvalinate | 15.6 | 50 | 520 > 208 | 21 | 520 > 181 | 44 | 5 |
| Linuron | 11.8 | 45 | 249 > 160 | 24 | 249 > 182 | 22 | 5 |
| Malathion | 12.3 | 50 | 331 > 99 | 28 | 331 > 127 | 14 | 10 |
| Metalaxyl | 11.0 | 50 | 280 > 220 | 19 | 280 > 192 | 24 | 5 |
| Methamidophos | 0.9 | 55 | 142 > 94 | 30 | 142 > 125 | 30 | 20 |
| Methomyl | 5.8 | 50 | 163 > 88 | 12 | 163 > 106 | 14 | 5 |
| Metolachlor | 12.2 | 40 | 284 > 252 | 20 | 284 > 176 | 34 | 5 |
| Metribuzin | 8.7 | 40 | 215 > 187 | 24 | 215 > 84 | 26 | 5 |
| Nicosulfuron | 10.1 | 50 | 411 > 182 | 25 | 411 > 213 | 23 | 5 |
| Novaluron | 14.3 | 100 | 493 > 158 | 23 | 493 > 141 | 50 | 5 |
| Parathion | 13.2 | 60 | 292 > 236 | 20 | 292 > 264 | 14 | 5 |
| Penthiopyrad | 12.8 | 60 | 360 > 276 | 20 | 360 > 177 | 40 | 5 |
| Permethrin | 15.1 | 40 | 408 > 183 | 24 | 408 > 355 | 12 | 5 |
| Phorate | 12.8 | 40 | 278 > 75 | 18 | 278 > 199 | 14 | 10 |
| Phosmet | 11.7 | 60 | 318 > 160 | 14 | 318 > 133 | 48 | 5 |
| Pinoxaden | 13.2 | 70 | 401 > 317 | 36 | 401 > 57 | 42 | 5 |
| Propiconazole | 12.8 | 80 | 342 > 159 | 34 | 342 > 69 | 36 | 10 |
| Propoxur | 9.2 | 50 | 210 > 111 | 14 | 210 > 93 | 30 | 5 |
| Propyzamide | 11.5 | 70 | 256 > 190 | 20 | 256 > 173 | 32 | 5 |
| Pyraclostrobin | 13.2 | 50 | 388 > 194 | 16 | 388 > 163 | 23 | 5 |
| Pyrazon | 7.1 | 50 | 222 > 104 | 30 | 222 > 77 | 50 | 10 |
| Pyrimethanil | 11.4 | 40 | 200 > 107 | 32 | 200 > 82 | 34 | 10 |
| Pyroxsulam | 10.4 | 50 | 435 > 195 | 35 | 435 > 258 | 44 | 10 |
| Quizalofop-p-ethyl | 13.9 | 100 | 373 > 299 | 18 | 373 > 271 | 33 | 5 |
| Rimsulfuron | 11.1 | 50 | 432 > 182 | 26 | 432 > 325 | 20 | 5 |
| Simazine | 9.2 | 40 | 202 > 132 | 26 | 202 > 124 | 24 | 5 |
| Tebuconazole | 12.8 | 60 | 308 > 70 | 42 | 308 > 125 | 44 | 20 |
| Terbufos | 13.7 | 50 | 289 > 103 | 12 | 289 > 57 | 32 | 5 |
| Thiabendazole | 9.8 | 50 | 202 > 175 | 34 | 202 > 131 | 42 | 10 |
| Thiacloprid | 9.1 | 65 | 253 > 126 | 28 | 253 > 90 | 49 | 5 |
| Thiophanate-methyl | 9.3 | 70 | 343 > 151 | 26 | 343 > 311 | 16 | 5 |
| Tralkoxydim | 14.4 | 70 | 330 > 284 | 18 | 330 > 138 | 26 | 20 |
| Triallate | 13.7 | 70 | 304 > 143 | 34 | 304 > 86 | 23 | 10 |
| Tribenuron methyl | 12.3 | 70 | 396 > 155 | 22 | 396 > 181 | 24 | 10 |
| Trifloxystrobin | 13.8 | 60 | 409 > 186 | 23 | 409 > 206 | 20 | 5 |
| Triticonazole | 12.0 | 50 | 318 > 70 | 38 | 318 > 125 | 40 | 20 |
| Velpar | 9.5 | 60 | 253 > 171 | 22 | 253 > 71 | 40 | 5 |
| malathion-d6 | 12.2 | 50 | 337 > 127 | 17 | 337 > 291 | 11 | n/a |

RT = retention time (minutes)

DP = declustering potential (volts)

CE = collision energy (electron volts)

LOQ = limit of quantitation (ng/g)

**Supplementary Table 2.** Oral acute LD_50_ values for *Apis mellifera* for pesticides used in pollen ball hazard quotient (HQ) calculations

| Pesticide | Category | Oral acute LD_50_ value for *A. mellifera* (μg/bee) |
| --- | --- | --- |
| Boscalid | Fungicide | >166 |
| Chlorpyrifos | Insecticide | 0.15 |
| Cyhalothrin lamda | Insecticide | 0.027 |
| Cyprodinil | Fungicide | 112.5 |
| Deltamethrin | Insecticide | 0.07 |
| Fludioxonil | Fungicide | >100 |
| Pyraclostrobin | Fungicide | >110 |
| Velpar (hexazinone) | Herbicide | 60* |

*Unknown mode acute LD_50_ for *A. mellifera*
